# Dehydroepiandrosterone (DHEA) Supplementation in Rheumatic Diseases: A Systematic Review

**DOI:** 10.31138/mjr.20230825.dd

**Published:** 2023-08-25

**Authors:** Thelma L. Skare, Elizabeth Hauz, Jozélio Freire de Carvalho

**Affiliations:** 1 Rheumatology Unit, Hospital Evangélico Mackenzie, Curitiba, PR, Brazil,; 2 Biomedicine Student, Instituto Brasileiro de Medicina e Reabilitação (IBMR), Rio de Janeiro, Brazil,; 3 Núcleo de Pesquisa em Doenças Crônicas não Transmissíveis (NUPEN), School of Nutrition from the Federal University of Bahia, Salvador, Bahia, Brazil

**Keywords:** dehydroepiandrosterone, DHEA, rheumatic diseases, rheumatoid arthritis, dermatomyositis, fibromyalgia, Sjögren’s syndrome

## Abstract

**Background:**

Dehydroepiandrosterone (DHEA) is an adrenal hormone used to treat rheumatic conditions such as systemic lupus erythematosus (SLE), Sjogren’s syndrome (SS), rheumatoid arthritis (RA) with controversial results.

**Aim:**

To review the results of DHEA use in rheumatic diseases.

**Methods:**

PubMed, Scielo, Scopus, and Embase databases were systematically searched for articles on the treatment of rheumatic diseases with DHEA between 1966 and April 2023.

**Results:**

Twenty-one studies were identified: 13 in SLE, 5 in SS, 2 in RA, and 1 in fibromyalgia. DHEA use in SLE has shown a mild to moderate effect on disease activity, a positive effect on bone mineral density (BMD), and improved fatigue. The studies on SS showed a decrease in symptoms of dry mouth, but its performance did not differ from placebo in disease activity. In RA, a questionable effect on disease activity was noted. The only study on fibromyalgia failed to show any improvement. The drug was well tolerated; mild androgenic effects were the most common complaints.

**Conclusion:**

DHEA seems to have a place in SLE treatment, where it improves BMD and disease activity. The use in RA, SS, and FM is questionable.

## INTRODUCTION

The immune and neuroendocrine systems are closely related, and this interconnection has reciprocal repercussions. If by one side, adrenal steroids may modify the immune cells functioning and therefore alter the progression of autoimmune diseases; on the other, pro-inflammatory cytokines such as IL (interleukin)-1B, TNF (tumoral necrosis factor)-α, and IL-6 stimulate the secretion of the corticotrophin-releasing hormone by the hypothalamus, promoting the secretion of adrenal cortex hormones such as cortisol and DHEA.^1–3^

Dehydroepiandrosterone (DHEA) is a weak androgen secreted by the adrenal gland’s zona reticularis. It is considered the most abundant steroid hormone in the plasma; it is a precursor of sex hormones: androgens and estrogens.^4^

Dexamethasone and other glucocorticoids reduce serum levels of DHEA by suppressing the ACTH release.^5^ Administration of DHEA has been shown to improve animal-induced arthritis.^6–8^ This compound has anti-inflammatory and immunological properties; it inhibits the production of pro-inflammatory cytokines blocking the nuclear factor-kappa B (NF-kappa B) activation and increases the ratio of Th1/Th2 cytokines production.^9,10^ Furthermore, it has anabolic properties in muscles, bones, and endothelium.^11^ Its supplementation has considerable effects on mood, well-being, and sexuality, improving the quality of life in patients with adrenal insufficiency and healthy elderly individuals.^12,13^

Low levels of DHEA have been noted in systemic lupus erythematosus (SLE), rheumatoid arthritis (RA), and Sjogren’s syndrome (SS), suggesting a possible role for this steroid hormone in the treatment of autoimmune disorders.^14^ In rheumatic diseases, DHEA administration has been used in SLE, RA, SS, and fibromyalgia (FM), with results that are reviewed in the present paper.

## METHODS

### Literature review

This article proceeded an extensive systematic search of articles published in the following four databases: PubMed/MEDLINE, EMBASE, Scopus, and Scielo from 1966 to April 2023 using the following MeSH entry terms: “dehydroepiandrosterone “ OR “DHEA” OR “prasterone” AND “rheumatic” OR “rheumatologic” OR “systemic lupus erythematosus” OR “antiphospholipid syndrome” OR “vasculitis” OR “juvenile idiopathic arthritis” OR “fibromyalgia” OR “rheumatoid arthritis” OR “Sjögren’s syndrome” OR “myositis” OR “systemic sclerosis” OR “spondylarthritis” OR “gout. We used equivalent strategies in other databases. No language restrictions were applied. The authors followed PRISMA guidelines.^15^ Eligibility criteria were human studies, observational, randomised controlled trials or non-randomised, cross-sectional, and case series. All with a prospective design. Animal experiments, *in vitro* studies, revisions, meta-analysis, and opinion papers were excluded. A standardised form to extract the following information from relevant articles was designed: authors, year of publication, number of studied patients, demographic data, disease duration, study follow-up, DHEA posology, outcomes, and side effects. The results were synthesised in two tables and no meta-analysis process was used.

## RESULTS

**Figure 1.**
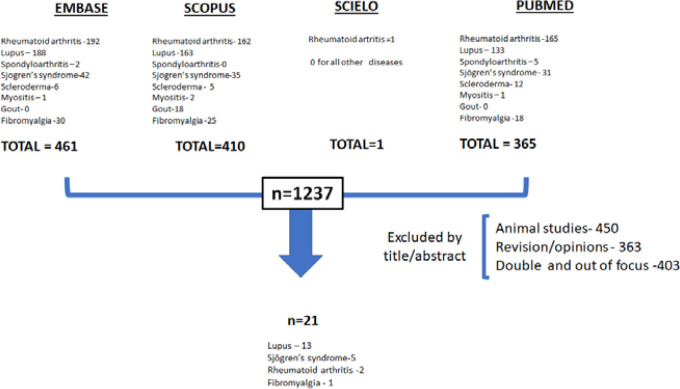
Search results.

Searching results are illustrated in **Figure 1**. Twenty-one studies were identified, most of them in SLE (13/21 or 61.9%), followed by SS (5/21 or 23.8%), two in RA (2/21 or 14.2%), and one in fibromyalgia (1/21 or 4.7%). No studies in systemic sclerosis, myositis, gout, and spondylarthritis were identified.

**Table 1** displays the SLE studies that encompass the observation of 1,119 individuals; all of them were done in females except for the study by van Vollenhoven et al.,^16^ which also studied males. The four main outcomes evaluated in this context were: disease activity,^16–21^ symptoms,^20,22,23^ serological markers,^21,24^ and bone mineral density (BMD).^16,23,25,26^ Most of the reports on the DHEA effect in SLE disease activity showed a good but modest response, with reduction or stabilization in disease activity,^16–19,21^ and the possibility of decreasing the prednisone requirement.^17,18,27^ Only in the small study by Marder et al.^28^ with 13 patients, improvement in disease activity measured by the SLEDAI could not be proved. Regarding the effects of DHEA on BMD, the results appear to be positive in the studies by Sanchez-Guerreiro et al.^26^ and Mease et al.^25^ as well as the effects on fatigue and general well-being.^20,22,23^ Nordmark et al.^23^ also studied the effects of DHEA on sexual performance and observed some improvement. The study in the lipid panel showed a reduction of HLDc in at least three studies,^21,23,28^ pointing to the fact that the DHEA effects on the lipid profile may not be beneficial. Most studies in SLE used supraphysiological doses of DHEA (200–100 mg/day);^17–22,24–27^ only one of them, by Nordmark et al.^23^ evaluating parameters of quality of life, used small doses: 20–30mg/day. The drug was generally well tolerated, with mild side effects related to the androgenic action of this compound: acne and hirsutism. Although some serious complications were related during some trials,^16, 20,26^ they appear to be mostly due to the activity of lupus itself.

In SS, the five studies encompassed 240 individuals, all females, and showed modest results.^29–33^ (**Table 2**). Some improvement in dryness, fatigue, and quality of life was observed without differences compared to placebo.^29–31^ Also, the two studies on RA were not reassuring;^34,35^ both had a small number of patients (one with 11 and the other with 46 patients), and one used a low dose (50 mg/day) while the other used supraphysiological dose (200mg/day) of DHEA. The use of supraphysiological doses had led the RA patients to achieve ACR of 20% in 18% of patients,^35^ while the other did not differ from placebo.^34^ The only study on fibromyalgia failed to show any benefit.^5^ (**Table 2**).

**Table 1.** Studies on the use of dehydroepiandrosterone (DHEA) in systemic lupus erythematosus (SLE).

**Author, reference**	**Study design**	**N**	**Age (years) /gender**	**Disease duration(years)**	**DHEA dose, (mg/day)/treatment duration**	**Primary outcomes**	**Outcome**	**Side effects**										
van Vollenhoven et al., 1994.^17^	Open, non-controlled study.	10	NA 100% females	NA	200mg	SLEDAI changes	↓ SLEDAI score, improvement in physician’s overall assessment, ↓ prednisone requirement. 3/10 patients with significant proteinuria, 2 showed marked and 1 modest reduction.	Mild acne.										
van Vollenhoven et al., 1995.^18^	Double-blind, placebo-controlled, randomised clinical trial.	28	NA 100% females	NA	200mg 3 months	SLEDAI changes	↓SLEDAI score, Improvement in patient’s and physician’s overall assessment of disease activity, ↓ prednisone dosage. Lupus flares more frequent in placebo group. (P = 0.053)	Mild acne										
Barry et al., 1998.^19^	Open prospective non-controlled trial.	23	100% females	NA	50mg initial dose, could be ↑ to 600mg/day. 6 months	SLEDAI, SLAM changes	↓SLEDAI, SLAM and patient VAS score. 10/23 achieved remission.	Acne, hirsutism										
van Vollenhoven et al, 1999.^16^	Double-blinded, placebo-controlled, randomised clinical trial for 6 months, followed by a 6-month open label period.	19	37 84% females (Patients with severe and active SLE)	NA	Double-blind phase- 200mg DHEA vs placebo Open-label phase 200mg/d DHEA	Stabilisation of the major lupus manifestations at 6 months.	Stabilisation of the major SLE manifestation (nephritis, haematological lupus, or serositis): DHEA: 7/9 Placebo: 4/10 (P<0.10). ↓ SLEDAI score: DHEA group: −10.3±3.1 Placebo: −3.9±1.4 (P<0.07). Bone mineral density DHEA: maintained Placebo: reduced	Serious adverse events: Double- blind phase: DHEA- 1 death; Placebo: deep vein thrombosis Fever + leukopenia Open-label phase SBO- 5 times in 1 patient Renal failure – 4 patients Sepsis- 1 patient Mild adverse events (+ in the DHEA group) Mild acne Altered menses										
Petri et al., 2002.^27^	Double-blind, randomised, placebo-controlled trial.	191	40, 100% females using 10–30mg/prednisone/day.	NA	100mg or 200mg 7–9 months	To determine whether DHEA would allow sustained reduction in corticosteroid.	↓ of prednisone to ≤7.5mg/day for at least 2 months in: 29% placebo, 38% DHEA 100mg, 51% DHEA 200mg. (Placebo vs 200mg/day with *P=*0.031).	Withdrawals due to side effects: 5% placebo, 6% in 100-mg DHEA ; 9% in 200-mg DHEA Acne, hirsutism, menstrual abnormalities, abdominal pain, headaches, insomnia.										
Chang et al., 2002.^20^	Multicentre randomised, double-blind, placebo-controlled trial.	113	NA, 100% females	NA	200mg; 24 weeks	SLAM changes	• SLAM - equal both groups Patients with flare: 16% DHEA vs. 33.9% placebo (*P=*0.044 ). Improvement in patient’s global assessment.	≥1 serious adverse events (secondary to LES flare): 7/61(11.5%) DHEA 18/59 (30.5%) placebo (*P=* 0.010). Acne.										
Chang et al., 2004.^24^	Double blind, randomised, placebo-controlled study.	30	32.5; 100% females	NA	200mg 24 weeks	Mean interleukin levels changes	No changes in IL1β ↓ in IL-10	NA										
Petri et al., 2004.^21^	Double-blind, randomised, placebo-controlled multicentre trial.	381	43.7; 100% females with active SLE	ND	200mg; up to 12 months	Lupus improvement or stabilisation without clinical deterioration.	Improvement or stabilisation without clinical deterioration: 58.5% - DHEA 44.5% - placebo (*P=*0.017 ) ↓ HDL-c, triglycerides, and C3 complement		DHEA	Placebo
								Acne	33%	14%
								Hirsutism	16%	2%
								Myalgias	22%	36%
								Oral stomatitis	15%	23%
Mease et al., 2005.^25^	Randomised, double-blind trial.	55	43.5 100% females receiving ≤10mg/prednisone/day- for ≥6 months	NA	200mg 1 year	BMD changes	BMD lumbar spine ↑ 1.7±0.8% - DHEA ↓ 1.1±0.5% placebo BMD hip ↑ 2.0±0.9% - DHEA ↓ 0.3±0.4% - placebo	NA										
Nordmark et al., 2005.^23^	Double-blind, randomised, placebo-controlled study for 6 months followed by a further 6 months of open labelled DHEA treatment for all patients.	41	20 to 65 100% females	NA	≤45 years of age-30mg ≥46 years of age-20mg 12 months	Health-related quality of life score improvement.	Improvement in SF-36 “role emotional” and HSCL-56 total score. Improvement in McCoy’s Sex Scale. DHE↓ HDL-c; ↑ insulin-like growth factor I and haematocrit. No effects on BMD or disease activity.	↑ hormonal body hair score (mild)										
Hartkamp et al., 2010.^22^	Randomised controlled trial.	60	43 (21–71) 100% females (with inactive SLE)	12.5 (2–32)	200mg 12 months with treatment + 6 months after cessation	General fatigue, depressive mood, mental well-being and physical functioning changes.	Improvement in general fatigue and mental wellbeing in relation to baseline equally in placebo and treatment groups.	Androgenic effects: 5 (16.6%) DHEA vs. 2 (6.6%) placebo.										
Marder et al. 2010.^28^	Double-blind placebo-controlled crossover trial.	13	37.7; 100% pre-menopausal females.	13 ± 7.9	200mg 22 weeks	Endothelial function, and circulating apoptotic endothelial cells (CD146_AnnVþ_), as well markers of bone metabolism. changes	↑ RANKL; ↓• HDL-c; Trend towards impairment of endothelial function; No differences: SLEDAI, CD146^AnnV+^ cells, or RANKL/osteoprotegerin.	None										
Sánchez-Guerrero et al. 2008.^26^	Double-blind phase → 200mg DHEA or placebo, followed by: Open-label phase → 100mg or 200mg DHEA.	155	NA, 100% females receiving glucocorticoid (60% post-menopausal)	9 years	200mg or placebo - 6 months in the double-blind phase; 100mg or 200mg - 12 months in the open label phase	Prevention of BMD loss	Double-blind phase- increase in BMD at the lumbar spine in DHEA group Open-label phase – increase in BMD in lumbar spine in a dose dependent manner. No changes in hip BMD	Double blind phase- similar in the DHEA and placebo group - except for acne and hirsutism. Interaction with warfarin-↑INR 4 deaths: Double-blind phase (n=2) both in the placebo group. Open label phase (n=2)- pulmonary embolism: placebo → 100 mg/day; “atherosclerosis”: 200 mg/day → 100 mg/day.										

N: number; SLAM: systemic lupus activity measure; BMD: bone mineral density; VAS: visual analogic scale; HDL-c: high density lipoprotein-cholesterol; SF-36: Short Form (36) Health Survey; HSCL: Hopkins Symptom Check List; SLEDAI: SLE disease activity index; RANKL: receptor activator for nuclear factor kB ligand.

**Table 2.** Studies on DHEA use in rheumatoid arthritis, Sjögren’s syndrome, and fibromyalgia.

**Author, reference**	**Study design**	**N**	**Age (years), gender**	**Rheumatic disease**	**Disease duration**	**DHEA dose (mg/day) Treatment duration**	**Outcome**	**Side effects**
Giltay et al., 1998.^35^	Open-label trial	11	52 (50–75) 55% females (post-menopausal)	RA	13 (4–38) years	200mg 16 weeks	- 18% improved ACR 20% and EULAR response criteria. - ↓ IFN, IL-2 and IL-4	9% had mild acne.
Sandoughi et al., 2020.^34^	Randomised placebo-controlled trial	46	36 100% females (pre-menopausal)	RA	5.79 years	50mg 12 weeks	- ↑ in QoL and ↓ DAS-28 and VHS in relation to baseline (p>0.05).	Equal in DHEA and placebo
Pillemer et al., 2004.^29^	Double blind, randomised, placebo-controlled trial	28	53.5 100% females	SS	NA	200mg 24 weeks	-7 DHEA vs 5 placebo achieved response criteria (p>0.05).	-16% dropout in DHEA and 7% placebo: - Acne, hirsutism, chills, arthralgia, nausea, abdominal discomfort, irritability, disseminated streptococcal infection and acute abdomen.
Porola et al., 2011.^32^	Single-blinded clinical trial using “up-and-down» method	12	55.7 (44–70) 100% females	SS	NA	50mg Two 4-month-long treatment periods	- ↑ serum DHEA and its correlated hormones. - No ↑ in salivary DHEA and DHT. - No clinical evaluation. - 2/12 ↓ Sjogren’s antibodies.	NA
Wirkki et al., 2010.^30^	Multicentre, investigator-based, powered, randomised controlled clinical trial	107	31–80 100% females	SS	NA	50mg 9 months	- DHEA and placebo improved equally fatigue and QoL	- 14% DHEA vs. 5% placebo: - Muscle cramps and skin maculae, sweating, itching, depression, acne, eczema, oral dryness, superficial thrombophlebitis, dental infection, migraine, headache, pelvic and heel pain, diarrhoea, hot flashes, increased tiredness or dreaming
Forsblad-d’Elia et al., 2009.^33^	Open prospective placebo-controlled trial.	33	60.7 ± 8.6 100% females (post-menopausal)	SS	6.0 ± 5.8	50mg 9 months	- ↓dry mouth -↑ serum DHEA and its metabolites.	1/33 (3%) nocturnal cramps.
Hartkamp et al., 2007.^31^	Randomised double-blinded placebo-controlled, counterbalanced, crossover trial	60	53.5 100% females	SS	7 years	200mg 18 months	- DHEA- and placebo improved fatigue, mental well-being, dry mouth, and depressive mood. - ↓ ESR in DHEA - ↓ dry eyes in placebo.	- Acne and hirsutism - 1 stopped participation: restlessness, malaise, night sweats, and skin rash; - 1 stopped: ocular pain and dryness, restlessness, and sleep disturbance. -1 patient: known to have mitral valve insufficiency, had heart failure 5 months after DHEA starting. -1 patient: lymphocytic interstitial pneumonitis.
Finckh et al., 2005.^5^	Prospective Randomised placebo-controlled trial	52	36–83 100% females (post-menopausal)	FM	11.5 years	50mg 3 months	No improvement: well-being, pain, fatigue, cognitive dysfunction, functional impairment, depression, or anxiety.	Greasy skin, acne, and hirsutism

DHEA: dehydroepiandrosterone; N: number, ACR: American College of Rheumatology; EULAR: European League Against Rheumatism; RA: rheumatoid arthritis; SS: Sjogren’s syndrome; FM: fibromyalgia; IFN: interferon; IL: interleukin; Qol: quality of life; DAS-28 ESR: disease activity score using 28 joints and erythrocyte sedimentation rate; N: not available.

## DISCUSSION

This study, reviewing the therapeutic effects of DHEA in rheumatic diseases, showed that, in SLE, this drug might have some indication. However, at the same time, in the other studied disorders: RA, SS, and FM, there is no evidence, so far, that this drug is helpful.

In SLE, the sex hormones play a role in the aetiopathogenesis of the disease; androgens and DHEA are found to be reduced in almost half of the patients,^36^ mainly in those with active disease.^37^ Moreover*, in vitro* studies have shown that this compound inhibits IL-6 and up-regulates IL-2, and studies in animal models of lupus; the administration of DHEA has led to a delay in the production of dsDNA autoantibodies as improved animal survival.^38,39^ In humans, it was not possible to prove that levels of the anti-dsDNA level changed with the DHEA treatment,^21^ but several reports describe some effect on disease activity,^16,17,19^ and that it decreases the number of flares.^16,20^. This drug has also shown a beneficial influence on the BMD of lupus patients, even in patients receiving low-dose glucocorticoids.^25^ This is probably due to its conversion of DHEA into androgens and oestrogens and its action regulating inflammatory cytokines and tissue growth factors.^40,41^

The influence of DHEA on the circulatory system needs more attention. DHEA was thought to have beneficial aspects from the cardiovascular point of view. Previously, Bonet et al.^42^ showed that it could avoid vascular remodelling by reducing cell proliferation and inducing apoptosis in proliferative cells. Lupus is known to be associated with endothelial dysfunction and premature cardiovascular disease, one of the leading causes of death in these individuals.^43^ In the general population, low levels of DHEA have been connected to amplified cardiovascular risk in men but not women.^44,45^ However, Marder et al.28 could not prove that the use of this drug was advantageous in this context, even suggesting an opposite effect, with a trend towards impairment of endothelial function measured by brachial artery flow-mediated dilatation. It is essential to consider that the study by Marder et al. was small and did not consider the possibility of DHEA reducing glucocorticoid requirement. The effect of DHEA on lipid profile is a matter of care. In the study by Petri et al.,^21^ HDLc and low-density lipoprotein (LDL) cholesterol levels were reduced with DHEA. The changes in LDL cholesterol were considered minimal but significant in the HDLc, and 26.6% of their patients had HDLc values under 40 mg/dL by the end of the study. In the general population, it has been shown that high DHEA in men may increase cardiovascular mortality;^46^ in females, no relationship between DHEA levels and coronary atherosclerosis could be found.^47^ So, more studies are needed in this context.

Reduced serum levels of DHEA have also been found in SS; however, the local levels of sexual hormones seem to influence the functioning of glandular tissue.^48^ Androgen and oestrogen are produced in local tissues from DHEA through an intracrine process that is unique to human beings.^48^ Testosterone and oestradiol regulate the expression of several genes in lacrimal gland tissue, with testosterone having a positive effect on dry eye, while the impact of oestrogen remains unclear.^48^ Interestingly, the work by Porola et al.^49^ showed that the DHEA treatment restored the plasma levels of androgens but did not correct the local deficiency in salivary glands, indicating a failure in the intracrine transformation of DHEA in SS.^32^ Forsblad-d’Elia et al.^3^ showed that the salivary flow rate did not increase with DHEA use despite improving subjective symptoms of oral dryness. General symptoms such as fatigue and feeling of well-being did not change in SS or FM patients.

Finally, two papers analysed patients with RA, including 57 patients. This disease is associated with low testosterone and DHEA serum levels in males and postmenopausal females,^49^ but the replacement showed a very modest effect in this setting.

In general, the drug was well tolerated in most cases. Side effects were uncommon; most of them were androgenic effects such as acne and hirsutism, which were considered mild.

The study strengths are: (1) inclusion of studies with patients with international criteria for rheumatic diseases; and (2) inclusion of all kinds of study designs on the use of DHEA in rheumatic disease. In this way, the authors believe that all published cases of DHEA in rheumatic patients were collected.

Limitations were also observed. None of the studies reviewed compared DHEA with classical treatments used in rheumatic diseases. Most of them had a low number of participants and short follow-ups. Therefore, future studies, including larger samples and more extended observations, are warranted. This would enable a better understanding of the DHEA treatment in rheumatic conditions. Another limitation is that there is the possibility that a few articles might be not collected by the search process in a systematic review.

## CONCLUSION

Few studies evaluate DHEA in rheumatological diseases, and only four such conditions were addressed in the literature: SLE, RA, FM, and SS. Most studies analysed in lupus demonstrated that DHEA use seems to have good effects on disease activity and BMD; results in RA, FM, and SS are disappointing. Side effects are mild and related to the androgenic effects. Therefore, DHEA may be a complementary option in patients with lupus, although future studies are indeed necessary to confirm these findings.
